# Effectiveness of community engagement in snakebite prevention and proper first aid practices: A community trial in rural Bangladesh

**DOI:** 10.1371/journal.pntd.0014180

**Published:** 2026-05-18

**Authors:** M. A. Faiz, Chowdhury Farheen, Rumana Rashid, Farhana Jahan, Abdullah Abu Sayeed, Aniruddha Ghose, Md. Robed Amin, Abu Shahin Mohammed Mahbubur Rahman, F. M. Atiqur Rahaman, Chinmaya Howlader, Sayra Khan, Nusaer Chowdhury, Geeta Rani Debi, Md. Shahidur Rahman, A. K. M. Fazlur Rahman

**Affiliations:** 1 Toxicology Society of Bangladesh, Chattogram, Bangladesh; 2 Dev Care Foundation, Chattogram, Bangladesh; 3 Centre for Injury Prevention and Research, Bangladesh, Dhaka, Bangladesh; 4 Bangladesh Institute of Tropical and Infectious Diseases, Chattogram, Bangladesh; 5 Chittagong Medical College, Chattogram, Bangladesh; 6 Directorate General of Health Services, Dhaka, Bangladesh; 7 Rajshahi Medical College Hospital, Rajshahi, Bangladesh; 8 Patuakhali Medical College Hospital, Patuakhali, Bangladesh; 9 Kalapara Upazilla Health Complex, Patuakhali, Bangladesh; 10 Shibganj Upazilla Health Complex, Chapainawabganj, Bangladesh; 11 Eastern Mediterranean Public Health Network, Dhaka, Bangladesh; LSTM: Liverpool School of Tropical Medicine, UNITED KINGDOM OF GREAT BRITAIN AND NORTHERN IRELAND

## Abstract

**Background:**

Snakebite is a neglected tropical disease with significant public health implications, especially in rural Bangladesh. Studies to identify an effective community-based intervention to address this underappreciated problem are scarce. This study evaluates the effectiveness of community engagement and enhanced health literacy for snakebite prevention and increased appropriate first aid practices utilizing existing health care system in rural communities of Bangladesh.

**Methodology and principal finding:**

This quasi experimental study was conducted in two rural sub-districts of Bangladesh. Educational sessions, visual aids, and culturally tailored community engagement activities were implemented in intervention area. Baseline and endline surveys were conducted to evaluate the effectiveness of the intervention in terms of outcome on preventive measures, first aid knowledge, and practices by the community people. In the intervention area, preventive knowledge improved modestly from 9.2% to 12% (OR: 1.38, 95% CI: 1.09–1.74), and first aid knowledge rose from 50% to 73% (OR: 2.62, 95% CI: 2.25–3.06). Positive practices increased significantly from 18% to 22% (OR: 1.24, 95% CI: 1.04–1.49). The incidence rate of snakebite increased by 10.6% in the control area while it dropped by 43.4% in the intervention area (from 294.4 to 166.5 per 100,000).

**Conclusion:**

The incidence of snakebite was significantly decreased by community-based initiatives that improved the knowledge and practice of community people. Tailored education, community engagement, and culturally sensitive strategies were key to success. However, gaps in sustaining positive practices highlight the need for ongoing support. These findings provide a replicable model for addressing snakebite challenges in resource-limited settings.

## Author summary

Snakebites continue to be a major health threat in many rural communities, yet awareness about prevention and proper first aid remains limited. Unlike previous studies that focused solely on measuring snakebite incidence, this research evaluates the effectiveness of community engagement with tailored health literacy strategies. This study was conducted in two areas: Kalapara from Patuakhali district, which was the intervention site, and Shibganj from Chapainawabganj district, which was the control site. Different intervention packages, such as educational sessions among different stakeholders (teachers, students, imams, and fishermen), courtyard meetings, and street shows, were delivered to make the community people aware about the preventive and first aid measures for snakebite. Additionally, the health care professionals, including the community health care providers, were trained and involved in the intervention process. Then we measured the changes in knowledge and practice behavior across the study sites. Although our efforts reached a wide audience, the overall improvement in knowledge and practices was smaller than we had hoped. This experience taught us that changing deep-rooted behaviors requires more than just awareness, it takes time, trust, and sustained involvement. Our findings highlight that while community-based interventions hold promise, stronger strategies and longer-term commitment are needed to bring real change.

## Introduction

Snakebite is an old, important, time-critical medical emergency mostly happening in the rural tropics. The World Health Organization (WHO) estimated 5.4 million bites, 1.8-2.7 million envenomation cases, and 81,410–137,880 deaths per year globally [1]. Bangladesh has one of the highest rates of snakebite in the world, with an estimated 623/100,000 cases and (~~6000 deaths) per year among the rural community [2]. Snakebite is considered an occupational hazard among the high-risk groups like cultivators, livestock farmers, fisherman and other agricultural workers during day-to-day activities of their livelihood while working barefoot and barehanded, having little knowledge and avenue for protection [3–5].

Of late, the WHO recognized the burden of snakebite by classifying it as a neglected tropical disease in 2018 and quickly acted upon it to develop the ‘WHO strategy for prevention and control of snakebite envenoming’ in 2019 [6,7]. The global strategy and regional action plan (WHO, SEARO) developed were aimed to reduce snakebite-related mortality and disability by at least 50% by 2030 through a number of strategies, notably through empowering and engaging the communities ‘to encourage better education about risks and avoidance to prevent snakebites and health care-seeking behavior’ [8,9]. The WHO Regional Office for South-East Asia, in the guidelines for the management of snakebites, also provided recommendations for community education for prevention and first aid [9]. In many affected countries, including Bangladesh, there is no dedicated public health program for mitigation of snakebite, and the national strategy is yet to be formulated, but India is at the forefront of such novel action [10,11]. The basic knowledge and practice of the community for prevention and first aid following snakebite has been found to be variable but far from optimal, and on some occasions harmful, for example, sharing poultry and food grains in the bedroom, applying a tight tourniquet, giving an incision following the bite, and relying on traditional healers for seeking care [12–15]. The baseline survey before implementing community interventions on health literacy among the rural Bangladeshi population on first aid measures and prevention of snakebite found a big gap to address [16].

Community engagement is a well-known method for addressing public health problems used before, notably for a number of neglected tropical diseases [17,18]. According to WHO, it has been defined as a process of developing relationships that enable stakeholders to work together to address health-related issues and promote well-being to achieve positive health impact and outcomes [19]. It often described as informing, consulting, involving, collaborating and empowering the community about informed decision making [20]. Fundamentally, community engagement essentially allows for changes in community behaviors, policies, programs, and settings [19]. Currently, there is no consensus about appropriate methods for engaging the community, and the WHO toolkit for community engagement for snakebite mitigation is yet to be available. Society-led community interventions to mitigate snakebite burden are a new concept provided by The SHE India [21]. As a part of providing community education on snakebite mitigation, the information, education and communication (IEC) materials were developed and used in some countries, but systematic study to see the impact of such community engagement is lacking or limited [22–25].

**Fig 1:**
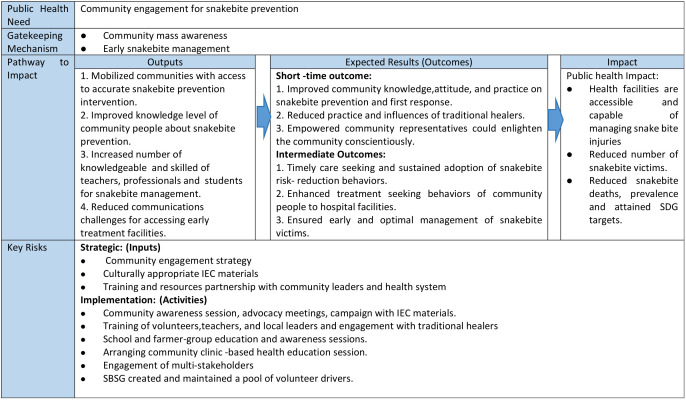
Theory of Change.

However, globally, community-based intervention trials or programs targeting snakebite prevention remain scarce. Multifaceted community education programs in India and Brazil have been advocated as powerful tools for mitigating snakebite envenoming, combining locally adapted materials and participatory approaches [26]. Additionally, in some parts of Africa, such as Eswatini (through community outreach that includes teaching preventive measures, myth-busting, and volunteer snake catchers) and Ghana, it has been demonstrated that community engagement can reduce risk [27]. A recent study conducted among Indonesian farmers found that digital health education could be effective in improving first aid measures [28]. However, these awareness programs/interventions were mostly either small-scale or poorly evaluated, or limited to awareness campaigns without rigorous measurement of behavior change. While previous studies in Bangladesh have primarily concentrated on documenting the incidence and burden of snakebite, there has been a notable gap in exploring proactive strategies to mitigate its impact. In particular, no prior initiatives have systematically assessed whether improving health literacy through community engagement can lead to better prevention and first aid practices. Therefore, to our knowledge, this is the first rigorously evaluated, large-scale quasi-experimental community-based snakebite prevention trial globally. By addressing both preventive behaviors and emergency response practices, this study aims to contribute critical evidence for developing sustainable, community-based solutions to reduce snakebite morbidity and mortality in similar low resource settings (Fig 1).

## Methods

### Ethics statement

Prior to participation, written informed consent was secured from each individual or their legally authorized representative. All eligible participants were asked to read and sign the Participant Information and Consent Forms outlining the scope of the study, the risks and benefits of participating in the study procedures and their role in the study. Ethical clearance was granted by the ethical review committee of the Bangladesh Medical Research Council (no. 506 23 10 2022).

### Study design, study setting

**Fig 2:**
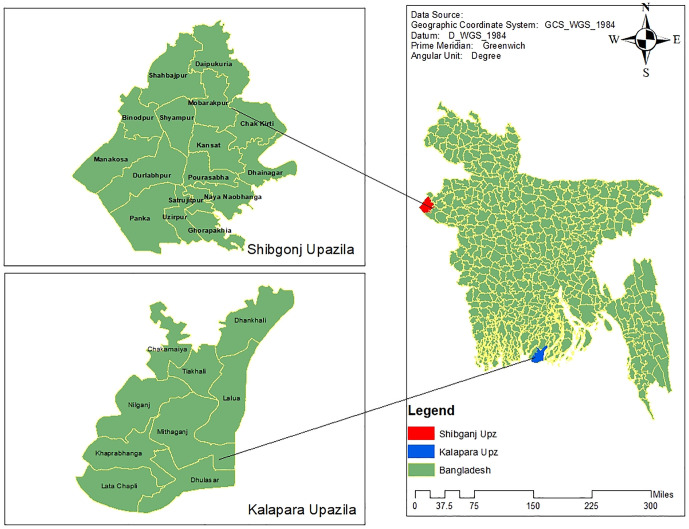
Map of the intervention and control area. Map Data Source is: Local Government Engineering Department (LGED) & Bangladesh Bureau of Statistics (BBS), 2025. Source Link: https://data.humdata.org/dataset/cod-ab-bgd; Government of Bangladesh, via LGED/BBS. Processed by OCHA for HDX.

This study is a quasi-experimental study conducted over a 24-month period, from September 2022 to August 2024. It took place in two rural communities of Bangladesh: Kalapara upazila of Patuakhali district which was our intervention group and Shibganj upazila (subdistrict) of Chapainawabganj district was our control group. Intervention area is situated on the bank of the Bay of Bengal with twelve unions and thirty-three community clinics. The community clinic infrastructure facilitates awareness of community-based health risks and ensures a comprehensive essential health care services to 6,000 individuals in remote communities. Similarly, the control area is located in the northernmost part of Bangladesh and surrounded by rivers, and consisted of fifteen unions (Fig 2).

The intervention and control areas were indeed selected purposively, mainly due to operational feasibility and the higher burden of snakebite cases in the intervention site compared to the control site [2]. While Shibganj Upazila was selected as the control site due to its broad socio-demographic comparability and geographic distance, which minimized the likelihood of contamination. To ensure baseline comparability**,** we used both the Population and Housing Census 2022 [29]. Census data confirmed that Kalapara and Shibganj were broadly similar in terms of household size, sex ratio, access to electricity, mobile phone ownership, and youth NEET rates (Table 1). We also took specific steps to minimize contamination between areas, such as choosing geographically distant subdistricts (separated by several hundred kilometers).

**Table 1:** Comparative table of Kalapara and Shibganj on key sociodemographic characteristics.

Indicators	Intervention area	Control area
Household size	4.00	3.9
Male: Female	1.02:1	1.01: 1
Ownership of mobile phone (%)	58.8	52.0
Access to electricity (%)	99.4	98.4
NEET youth population (%) *	39.9	39.63

*Proportion of youth (aged 15–24 years) not in education, employment or training

### Study participants

The eligibility criteria of the study participants were people residing in the same household for the last six months. Additionally, to select the primary respondent, the household heads were considered.

### Sample size

The sample size was estimated to detect a significant change in prevalence based on a two-sided significance level of 95%, a power of 80%, an allocation ratio of 1:1, and an expected effect size of 40% [30,31] with baseline and end line prevalence assumptions of 0.6% and 0.33%, respectively. This resulted in an estimated sample size was 120,315 individuals.

To achieve the sample size, 30,000 household from intervention and control area were surveyed. The baseline and end line survey were conducted following the intervention implementation using the same sampling strategy to evaluate changes in snakebite prevalence, knowledge, and preventive practices. However, to assess the knowledge and practices related to snakebite prevention and first aid measures, 10% of the total households were randomly selected.

### Sampling strategy

**Fig 3:**
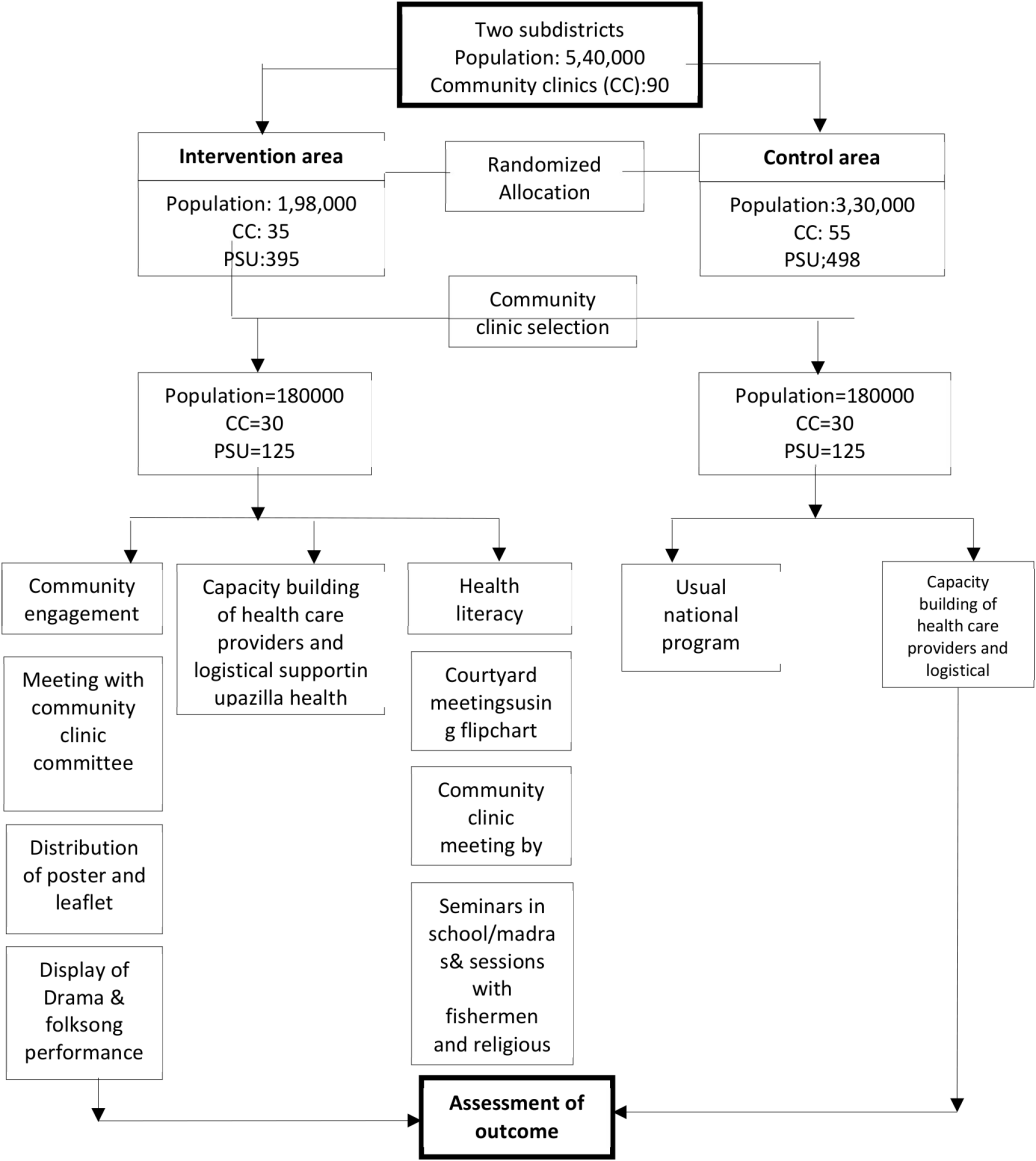
Methodological Framework.

A purposive selection strategy was employed to identify one subdistrict from the northern region and another from the southern region. Then we systematically identified community clinic catchment areas by compiling a list of all functioning community clinics. The eligibility criteria for clinic inclusion required that the community clinic demonstrate operational activity within the community, maintain a functional community clinic support group, and engage multipurpose health volunteers. Later, the community clinic catchment areas were then subdivided into primary sampling units (PSUs) to facilitate the household survey. A computer-generated randomization process was used to select 125 PSUs from each community clinic catchment area to reduce further selection bias. Within each PSU, 120 households were surveyed to estimate snakebite prevalence. However, systematic random sampling was applied to survey every tenth household for the assessment of knowledge and practices related to snakebite prevention and first aid measures (Fig 3).

### Procedure

#### Pre-intervention phase.

In the pre-intervention phase, a formative assessment was conducted to understand the overall situation as well as to understand the practices and beliefs for snakebite prevention within the community. This assessment acted as instrumental tool for the baseline questionnaire development. In this phase, we gathered sociodemographic characteristics, incident of snakebite and the knowledge, practice of snakebite prevention within the community members during November 2022 to January 2023

#### Intervention phase.

**Finalization of intervention-community engagement:** The community engagement approach incorporated multiple levels, including informing, consulting, involving, and empowering community members. Emphasis was placed on culturally tailored interventions that actively engaged the community and were adapted to the local cultural context, thereby enhancing their relevance and acceptability among participants. The design of the intervention was informed by the results of formative research, baseline surveys, and established guidelines, such as the National Guideline for Snakebite Management [32] and the WHO SEARO Guidelines (2016) [33]. The intervention package was improved through cooperative workshops with stakeholders and experts to successfully meet local needs. Additionally, necessary modifications were made in intervention delivery strategy after the midline process evaluation. Fidelity of the intervention was determined through midline process evaluation, including monitoring of planned vs. delivered activities (e.g., number of community meetings, seminars, conducted, and proportion of the target population reached). Supervisors used structured checklists to assess the quality and consistency of intervention delivery. For the assessment of the acceptability, we conducted two focus group discussions, and in-depth interviews with newly snakebite affected victims what caused the barriers/facilitators affected their treatment.

**IEC material development and demonstration:** Information, Education, and Communication (IEC) materials were developed by the research team by a series of workshop with clinicians, public health experts, and communication specialists. To guarantee cultural and contextual relevance, community health workers, educators, and local stakeholders also participated in the creation and presentation of these materials. Content was tailored through formative research, and pre-testing to ensure cultural appropriateness, clarity, and technical accuracy. Focus group discussions and in-depth interviews with community members, traditional healers, and health care workers helped identify misconceptions and locally relevant risk practices. Draft materials (posters, flipcharts, leaflets) were pre-tested among people with similar educational level to check clarity, language, illustrations, and acceptability. Revisions were made based on feedback. Messages were translated into Bangla and visuals were simplified to match literacy levels. The finalized materials were disseminated by field staffs, and facilitators of educational sessions through courtyard meetings, clinics, schools and community gatherings under supervision of project staff. (Insert S1–S4 Posters here).

**Implementation of interventions:** The implementation activity lasted from March 2023 to March 2024. Structured activities were incorporated through community engagement strategies to improve institutional and community awareness and readiness (Table 2). The Arnstein’s Ladder of citizen participation” could offer a useful lens to demonstrate how our intervention, engaged communities across different levels of participation [34]. The lower rungs of manipulation and therapy were absent, reflecting an intentional design to avoid symbolic or coercive involvement. Most health education initiatives such as flip-chart sessions, posters, folk songs, dramas, and social media content fell within the informing stage, ensuring wide knowledge dissemination. Activities like courtyard meetings, school seminars, and engagements with fishermen, imams, and bazaars reflected consultation, where community members were invited to receive information and share their views. However, decision-making authority remained within the research team. The signs of partnership were seen in the formation of snakebite support groups, collaboration with local NGO (CIPRB), and capacity building of healthcare providers, which fostered shared responsibility in awareness and preparedness. The intervention demonstrated a movement beyond tokenism toward partnership.

**Table 2:** Community-Based Intervention Activities.

Activity	Details
**Meeting in Community Clinics (CC)**	
i. Community Clinic Meeting	Trained community health educators to run sessions in their local areas.
ii. Community Clinic Bi-Weekly Sessions	CHCPs conducted bi-weekly health education sessions using flip charts.
iii. Courtyard Meetings	Trained 90 SBSGs on snakebite prevention and first aid; conducted 15 meetings per clinic area.
iv. Sessions at Upazila Health Complex	Health workers conducted 10–15-minute sessions using flip charts.
**Meeting with Teachers from Schools, Colleges, and Madrasas**	
i. Training of Teachers	Conducted training sessions for principals and teachers; distributed posters.
ii. Seminars for Students	Held seminars on snakebite prevention at 30 institutions.
iii. Scientific Fair and Art Competition	Organized a scientific fair and art competition on snakebite prevention.
**Awareness through Audio-Visual Content**	
i. Performance of Folk Song (‘Boyati’)	Local singers performed 30 shows in crowded areas.
ii. Display of Drama	Staged a drama on snakebite malpractice and referral; displayed at Upazila Health Complex and on social media.
**Poster and Leaflet Distribution**	Distributed 7000 posters and 21000 leaflets in schools, markets, and offices.
**Collaboration with Center for Injury Prevention and Research (CIPRB), Bangladesh**	Trained CIPRB “Achal” officers on snakebite prevention; conducted 170 sessions at parent meetings.
**Facebook Page Activity**	Shared educational content (posters, videos, dramas) on snakebite prevention via the page “সর্পদংশনবিষয়কসচেতনতা” কলাপাড়া”
**Video Display at UHC Intervention area**	Displayed two dramas on snakebite awareness (12-min and 1-min) every 15–20 minutes.
**Community Clinic Committee Meeting**	Arranged additional 30 meetings over four months to raise snakebite awareness.
**Health Education in Educational Institutions**	Conducted seminars/sessions in 10 institutions using posters.
**Health Education with Fishermen**	Conducted a session on snakebite prevention and first aid at Mohipur Fishery Ghat.
**Health Education in Local Bazars**	Conducted 4 sessions on snakebite prevention and first aid in bazaars.
**Health Education with Imams**	Conducted a session with 7–8 Imams, encouraging them to share messages during weekly prayers.

#### Post intervention.

The evaluation process followed both the same tool to enable a consistent comparison of changes over time. In this research, the health literacy status was measured through participants knowledge and practice of preventive and first aid measure. Specially, health literacy is operationalized by the degree to which individuals have the ability to find, understand, and use information and services to inform health-related decisions and actions for themselves and others [35]. The evaluation phase data collection took place from May 2024 to July 2024.

### Outcomes

**1. Knowledge of Preventive Measures (10 Questions):** Evaluated knowledge of precautions like wearing boots in the field, keeping the environment clean, using mosquito nets, and refraining from dangerous practices like reaching into holes or keeping crops in bedrooms (Table 3).

**Table 3:** Different types of questions asked to respondent.

Preventive Measure	First Aid	Practice
Using mosquito net while sleeping on floor^*^	Immobilize the bite site.	Using mosquito net
Being cautious while guarding the crop fields and sleeping at night	Immediate referral to nearest hospital.	Being cautious while guarding the crop fields and sleeping at night
Keeping the surrounding clean	Suck out blood from the bite site^*^	Keeping the surrounding clean
Using boots while working at fields	Using tourniquet at the bite site^*^	Using boots while working at fields
Be cautious while handling stacked materials	Incision at bite site^*^	Be cautious while handling stacked materials
During movement use torch in darkness	Applying ashes chicken wastage at bite site^*^	Look into the water cautiously while fishing
Do not store grains/crops in the bedroom	Visiting traditional healer^*^	Using torch in night
Tapping the floor with stick while walking	Casting mantras by traditional healer^*^	Tapping the floor with stick while walking
Do not put hand inside any hole		Do not store grains/crops/in the bedroom
Using mosquito net while sleeping on bed		Not keeping chickens, pigeons and ducks in the bedroom
		Do not put hand inside any hole

* is negative question, rest were positive question

**2. First Aid Knowledge (8 Questions):** Evaluated participants’ knowledge of proper reactions to snakebite, including immobilization of the bite site and immediate hospitalization. Using tourniquets, sucking blood, cutting, applying ashes, or seeking advice from traditional healers are examples of harmful practices that should be avoided (Table 3).

**3. Practice (11 Questions):** Assessed practical implementation of first-aid and prevention practices, such as regular use of mosquito nets, keeping the environment clean, moving carefully at night, and safe practices when farming or fishing (Table 3).

### Data collection

Data collectors holding at least a bachelor’s degree, were recruited following standard procedures for both baseline and end line data collection. In both phase, training of data collectors covered the research concept, objectives, key indicators, interview techniques, questionnaire formats, data collection, record-keeping, and confidentiality.

### Statistical analysis

Stata version 20 was used for the analysis of the data. Descriptive analysis was conducted to summarize the characteristics of the study population. Independent two-sample t-tests were performed to examine differences in mean scores between the two study areas for Preventive Measures, First Aid, and Practice. Additionally, Chi-square tests were used to assess associations between categorical variables. Logistic regression models were applied to evaluate the association between the dependent variables (Preventive Measures Knowledge, First Aid Knowledge, and Practice) and the study areas, adjusting for potential confounders. Three separate logistic regression models were conducted: one for Preventive Measures Knowledge (coded as 1 for Good Preventive Measures Knowledge and 0 for Poor Preventive Measures Knowledge), one for First Aid Knowledge (coded as 1 for Good First Aid Knowledge and 0 for Poor First Aid Knowledge), and one for Positive Practice (coded as 1 for Negative Practice and 0 for Positive Practice). Knowledge scores were deemed “Good” if participants obtained >5/10 in Preventive Measures Knowledge or >4/8 in First Aid Knowledge. A practice scores greater than 5/11 was considered “Positive,” while scores of ≤50% in any segment were classified as poor [36].

Visualizations included a confidence interval (CI) plot depicting changes in mean scores and a line and point chart previously uploaded, providing a graphical overview of trends. Statistical significance was determined at a p-value of < 0.05. During pre and post intervention data collection process, four supervisors were employed to ensure quality and compliance with study guideline.

## Results

In both study areas, 20,356 (36%) and 21,507 (36%) of the study population were under the age of 18, whereas 2987 (5.3%) from the intervention area and 3704 (6.2%) from the control area belonged to the > 60 age category. Both the study areas had equal male-female ratios (27,921 [50%] vs. 30,235 [50%]). Farming, fishing, and homemaking were more common in the intervention area (23,462 [48%] vs. 23,544 [45%]), while drivers, laborer and business occupations are higher in comparison area (11,697 [22%] vs. 8,807 [18%]). Additionally, 10686, 19% of individuals had no formal education, compared to 19373, 32% in the comparison site. However, in intervention area, 4,477 (8.0%) participants attained higher secondary education and above than the control area 5,406 (9.0%) (Table 4).

**Table 4:** Population characteristics of the intervention and control area.

Characteristic	Intervention area (Kalapara) N = 56,052^a^	Control area (Shibganj) N = 59,967^a^
**Age Group**		
<18	20,356 (36%)	21,507 (36%)
18-30	13,329 (24%)	13,061 (22%)
30-45	12,448 (22%)	12,877 (21%)
45-60	6,932 (12%)	8,818 (15%)
>60	2,987 (5.3%)	3,704 (6.2%)
**Sex**		
Female	28,131 (50%)	29,732 (50%)
Male	27,921 (50%)	30,235 (50%)
**Occupation**		
Farming/ Fishing/ Homemaker	23,462 (48%)	23,544 (45%)
Teacher/ Student/ Job Holder	15,009 (30%)	15,260 (29%)
Transport driver/ Laborer/ Business	8,807 (18%)	11,697 (22%)
Others	2,010 (4.1%)	2,346 (4.4%)
**Education**		
No Education	10,686 (19%)	19,373 (32%)
Primary/Religious Studies	24,138 (43%)	17,956 (30%)
Secondary	16,751 (30%)	17,232 (29%)
Higher Secondary and above	4,477 (8.0%)	5,406 (9.0%)

**Fig 4:**
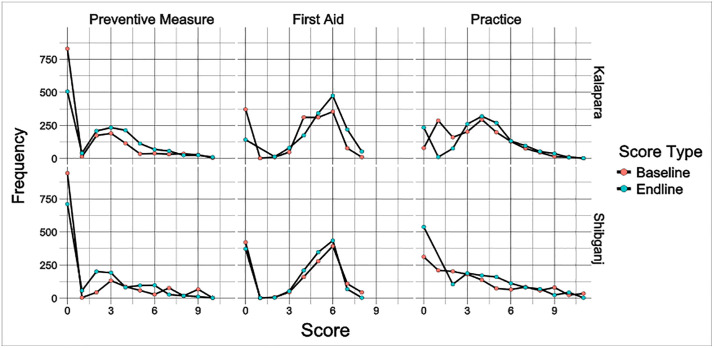
Score of Preventive Knowledge, First Aid and Practice in Intervention and Control area.

The line graph illustrates the changes in knowledge and practice scores for preventive measures and first aid methods of snakebite in the intervention and control area at baseline and endline. In the intervention area, there has been a noticeable shift towards higher scores across all domains. In the domain of first aid knowledge, intervention area demonstrated a broader knowledge score ranging from 3–6, while in the end line, most of the participants clustered around the score of 6. The practice scores in intervention area also showed improvement, with baseline scores centered around 3 and 4 shifting to higher scores at the end line, with an increase observed in the 4–5 range. The control area showed only minor improvements, with score distributions largely remaining concentrated in the lower ranges at both time points (Fig 4).

**Fig 5:**
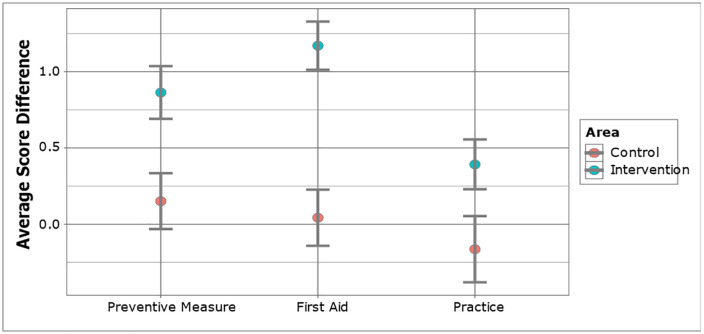
Score difference of Preventive Knowledge, First Aid and Practice in Intervention and Control area.

Fig 5 demonstrates that the Confidence Interval plot highlights significant improvements in the intervention area across all metrics: Preventive Measure (0.864, CI: 0.691–1.04), First Aid (1.17, CI: 1.01–1.33), and Practice (0.392, CI: 0.229–0.556), with confidence intervals excluding zero. In contrast, the control area showed minimal or non-significant changes, with slight increases in the Preventive Measure (0.152, CI: -0.0314–0.335) and First Aid (0.0429, CI: -0.141–0.227) and a decline in Practice (-0.163, CI: -0.380–0.0537) (Fig 5).

Table 4 shows, there is a significant difference were observed across all scores post-intervention: the average preventive measure score increased from 1.74 to 2.6, First Aid Score from 3.82 to 4.99, and Practice Score from 3.54 to 3.93 in the Intervention area (p < 0.001). In the Control area, no significant differences were noted, with scores remaining relatively stable across all categories (Table 5).

**Table 5:** Changes in mean score of Preventive knowledge, First aid and Practice in intervention and control area.

Characteristic	Intervention Area (Kalapara) Mean ± SD			Control area (Shibganj) Mean ± SD		
	Baseline N = 1,494^a^	Endline N = 1,497^a^	p-value^b^	Baseline N = 1,460^a^	Endline N = 1,493^a^	p-value^b^
Preventive Measure Score	1.74 ± 2.392	2.6 ± 2.433	**<0.001**	1.78 ± 2.759	1.93 ± 2.289	0.10
First Aid Score	3.82 ± 2.398	4.99 ± 2.001	**<0.001**	3.88 ± 2.663	3.91 ± 2.421	0.71
Practice Score	3.54 ± 2.213	3.93 ± 2.344	**<0.001**	3.32 ± 3.074	3.16 ± 2.937	0.15

^a^n (%),^b^Two sample t-test.

Table 5 summarizes the baseline and endline proportions, odds ratios (OR), and adjusted odds ratios (AOR) for good preventive measure knowledge, good first aid knowledge, and positive practice in Intervention area and Control area. Preventive Knowledge increased from 9.2% to 12% (OR: 1.38, AOR: 1.37, 95% CI), First Aid Knowledge from 50% to 73% (OR: 2.62, AOR: 2.66, 95%), and positive practice from 18% to 22% (OR: 1.24, AOR: 1.23, 95% CI: 1.03–1.48). In contrast, the Control area showed a decline in preventive knowledge (13% to 10%, OR: 0.77, AOR: 0.74, 95%), stability in First Aid Knowledge at 57% (OR: 1.03, AOR: 0.98, 95%), and a slight reduction in Positive Practice (24% to 22%, OR: 0.92, AOR: 0.89, 95%) (Table 6).

**Table 6:** Effectiveness of intervention over two phases in Intervention and Control area.

	Intervention Area (Kalapara)				Control area (Shibganj)			
Characteristic	Baseline N = 1,494^a^	Endline N = 1,497^a^	OR (95% CI)	AOR (95% CI)	Baseline N = 1,460^a^	Endline N = 1,493^a^	OR (95% CI)	AOR (95% CI)
Good Preventive Measure (Knowledge)	138 (9.2%)	184 (12%)	1.38 (1.09, 1.74)	1.37 (1.10, 1.70)	191 (13%)	155 (10%)	0.77 (0.61, 0.96)	0.74 (0.62, 0.93)
Good First Aid (Knowledge)	752 (50%)	1,088 (73%)	2.62 (2.25, 3.06)	2.66 (2.28, 3.10)	825 (57%)	853 (57%)	1.03 (0.89, 1.19)	0.98 (0.85, 1.14)
Positive Practice	275 (18%)	328 (22%)	1.24 (1.04, 1.49)	1.23 (1.03, 1.48)	346 (24%)	331 (22%)	0.92 (0.77, 1.09)	0.89 (0.75, 1.06)

^a^n (%), ^a^OR = Odds Ratio, AOR = Adjusted Odds Ratio, CI = Confidence Interval

Adjusting for Age Group, Sex, Education, Occupation

Table 6 compares snakebite incidence in the Intervention area and the Control area between baseline and endline. In the Intervention area, cases dropped from 165 to 98, with the incidence rate declined significantly from 294.4 to 166.5 per 100,000, a 43.4% reduction (95% CI: -43.58% to -43.30%). Conversely, in the Control area, cases rose from 55 to 61, with the incidence rate increasing from 91.7 to 101.5 per 100,000, a 10.6% rise (95% CI: 10.25% to 11.06%) (Table 7).

**Table 7:** Human snakebite incidence rate by upazila per 100,000 populations.

Characteristic	Intervention Area (Kalapara)			Control area (Shibganj)		
	Baseline	Endline	% of Change (Baseline vs Endline)	Baseline	Endline	%of Change (Baseline vs Endline)
Number of Snakebite Cases (N)	165	98		55	61	
Incidence Rate (Per 100000)	294.4	166.5	-43.4%	91.7	101.5	10.6%
CI (95%) (Per 100000)	252.7 342.9	136.6 203.0	-43.58% -43.30%	70.4 119.5	79 130.4	10.25% 11.06%

^a^n (%)

## Discussion

Death following snakebite is proportionate to the incidence/number of bites happening in the community besides improved management. Our findings from this community trial show that implementation of a package of interventions through community engagement reduces the incidence rate by 43% in the intervention area. First aid and prevention of snakebite in the intervention area showed notable improvements as a result of the community-based interventions. In the intervention area, snakebite cases and incidence rates significantly decreased, whereas in the control area, both metrics increased. Knowledge of both first aid and prevention significantly increased among the participants in the intervention area. The participants’ positive immediate response to snakebites and their use of prevention techniques also demonstrated small but steady improvement. These results show how well the interventions worked to reduce the risk of snakebite by combining behavior change, education, and active community involvement.

### Impact of intervention

The interventions at the intervention area led to a significant reduction in snakebite cases and incidence rates, reflecting the efficacy of community-based strategies in addressing snakebite, an important public health issue. The number of reported cases in the intervention area dropped from baseline to endline, corresponding to a 43.4% reduction in the incidence rate. In contrast, in the control area, there is an increase in snakebite cases of 10.6%. These findings highlight the impact of targeted interventions that combined education, behavior modification, and local engagement to foster preventive practices and reduce risks. The results are very important in an area where snakebites happen often, as they show that health education programs in rural Bangladesh can lower snakebite cases by increasing awareness and promoting safer behaviors [8,35]. Another study on newborn care in the same country emphasized the importance of community mobilization for improving health outcomes in resource-limited settings, further underscoring the necessity of localized, participatory approaches [17]. Several key drivers effectively addressed the community’s specific risk factors, leading to the success of the intervention in the intervention area. Enhanced preventive practices, such as using torches at night, avoiding the storage of poultry and food grain in bedrooms, and tapping the floor with sticks while walking, significantly reduced human-snake interactions, a primary cause of snakebite in rural areas. The intervention also fostered improved knowledge about snakebite prevention and first aid, empowering individuals with critical information to adopt safer behaviors and effectively manage emergencies. Moreover, the participatory approach of the intervention ensured high levels of community engagement, leading to widespread acceptance and adherence. These efforts align with findings from India and Sri Lanka, which indicated that health programs designed for specific cultures and led by the community are essential for creating lasting changes in health behaviors among high-risk groups [21,22,36]. In the Brazilian Amazon, a modest decrease in snakebite incidents has been found 3 years following a multifaceted program for community health education but not quantified in the paper by Vaiyapuriet et al. [22]. Collectively, these elements demonstrate how comprehensive, community-centered programs can mitigate the burden of neglected tropical diseases like snakebite.

### Improvement in preventive knowledge

One of the most notable outcomes of the interventions was the improvement in knowledge regarding various preventive measures. Educational sessions, such as bi-weekly health meetings at community clinics, courtyard gatherings, traditional songs, and interactive workshops, could achieve this. During these activities, emphasis was given to practical preventive measures, such as using mosquito nets, wearing boots while working in fields, and avoiding the storage of grains and poultry in bedrooms—practices that reduce the risk of snake encounters. Visual aids like flip charts, combined with the distribution of posters and leaflets, reinforced these messages and extended their reach beyond the immediate participants. In contrast, the control area experienced rather a decline in preventive knowledge, from 13% at baseline to 10% at end-line, which underscores the necessity of continuous education and active engagement to sustain awareness. This finding aligns with prior studies indicating that consistent reinforcement of knowledge is essential to maintaining and improving preventive behaviors [8,9]. A significant improvement in awareness in communities that attended the educational outreach program was found by the Madras Crocodile Bank Trust but not objectively measured/quantified [22].

### Significant gains in first aid knowledge

The improvement in first aid knowledge was even more striking in the intervention. In one area, the proportion of individuals with good knowledge of first aid practices rose dramatically from 50% at baseline to 73% at end-line (OR: 2.62, 95% CI: 2.25–3.06). Training sessions at community clinics and the sub-district hospital provided critical guidance on first aid measures, such as immobilizing the bite site, avoiding harmful practices like using tourniquets or making incisions, and seeking immediate medical care. Educational seminars in schools and colleges further amplified the reach of these messages, embedding correct first aid knowledge among younger generations. Culturally resonant activities, including “Boyati (folk)” song performances and drama displays, also played a pivotal role in addressing common misconceptions about first aid. By integrating these methods, the interventions ensured that the information was accessible, engaging, and relatable to diverse community members. Conversely, the control area exhibited no significant change in first aid knowledge, which remained at 57% from baseline to endline (OR: 1.03, 95% CI: 0.89–1.19). This lack of progress in the control area reflects the absence of structured interventions and highlights the need for proactive health education to counter harmful traditional practices, as documented in other rural settings with low resources in the region, including Bangladesh [12,13].

### Limited progress in positive practices

The improvement of practices at the intervention area was significant, even though it was modest over the 20-month period. Positive practices, such as wearing boots, using torches at night, and tapping the ground with a stick while walking, increased significantly. These behavioral changes were supported by activities like village market (‘bazar’) sessions, meetings with religious leaders, and parent education initiatives led by ‘Anchal’ officers of the Centre for Injury Prevention Research, Bangladesh (CIPRB). These approaches addressed cultural and social barriers, encouraging the community to adopt safer practices gradually. In contrast, the control area experienced a slight decline in positive practices, which underscores the challenges of promoting behavior change in the absence of targeted interventions. In a study conducted in Sri Lanka, a mismatch was found between knowledge and practice among the farmers; despite adequate knowledge, they do not practice appropriate prevention and first aid measures for snakebite [36]. Previous studies have similarly highlighted the difficulty of sustaining positive practices without continuous engagement and reinforcement [5,6].

The data reveal significant differences between the intervention and the control area in terms of age, gender, occupation, and education, which influenced the outcomes of snakebite prevention interventions. The intervention area’s younger demographic (32% aged 18–30 vs. 25% in the control area) facilitated greater engagement with educational activities, while the control area’s older population faced challenges in adopting new behaviors. Higher female participation in the intervention area (51% vs. 47% in the control area) amplified the impact of interventions due to women’s roles as caregivers and homemakers. Occupation patterns showed the intervention area’s high-risk farming and fishing occupations aligned with targeted preventive measures, whereas the control area’s diverse occupations like driving and business required tailored strategies. Also, the intervention area had a lower illiteracy rate (7.4% compared to 30% in the control area), which helped people adopt preventive and first aid practices more easily, highlighting the importance of using simple and culturally relevant strategies in communities with less education [9,18,22].

This study has important limitations. The intervention and control districts were purposively selected**,** which may have introduced selection bias and limits the generalizability of findings to other settings with different socioeconomic or ecological characteristics. Especially, the findings may not have been as comprehensive due to the exclusion of children and underrepresentation of diverse occupational groups. On the other hand, the study did not follow the same cohort over time but rather employed repeated cross-sectional surveys, which may have influenced the measurement of changes in snakebite-related knowledge. Furthermore, the outcomes were based on self-reported knowledge and practices, making them susceptible to recall and reporting biases that could have resulted in either underestimation or overestimation of the true effects. The main challenges we encountered during community engagement processes were resources and time constraints, managing expectation and conflicting priorities, and superficial enagement. Despite these limitations, the results provide a scalable framework for improving immediate care and prevention of snakebite in environments with limited resources. However, higher rungs of community engagement such as delegated power and citizen control were not achieved through this research but these activities laid the foundation for stronger, citizen-driven participation in future scale-up and sustainability efforts.

## Conclusion

While our findings indicate that community engagement can improve snakebite-related health literacy, scaling up such initiatives must be aligned with Bangladesh’s National Snakebite Strategy. The strategy already outlines a governance structure through the formation of a national steering committee, technical committee, working group, and district- and upazila-level snakebite committees, with clearly defined roles. At the grassroots level, Union Parishads and community clinic groups are mandated to include snakebite prevention and control in their regular meetings. Embedding community engagement interventions within this governance framework would ensure that efforts are not implemented in isolation, but are instead institutionalized and monitored through existing mechanisms. Operationalizing this approach would require the government to strengthen resource allocation and accountability, NGOs to support innovation in awareness and training methods, and community leaders to facilitate local ownership and cultural adaptation. Additionally, leveraging digital tools such as mobile-based surveillance and telehealth consultation could accelerate reporting and response at community level, complementing the existing structure.

## Supporting information

S1 Poster IEC material. (JPG)

S2 Poster IEC material. (JPG)

S3 Poster IEC material. (JPG)

S4 Poster IEC material. (JPG)

S1 Data. Baseline. (XLSX)

S2 Data. Baseline KAP. (XLSX)

S3 Data. Endline. (XLSX)

S4 Data. Endline KAP. (XLSX)
