# Supplementary figures and images for: Effectiveness of community engagement in snakebite prevention and proper first aid practices: A community trial in rural Bangladesh
Source: PLoS Negl Trop Dis. 2026 May 18;20(5):e0014180. doi: 10.1371/journal.pntd.0014180 (PMC13262932; doi:10.1371/journal.pntd.0014180)

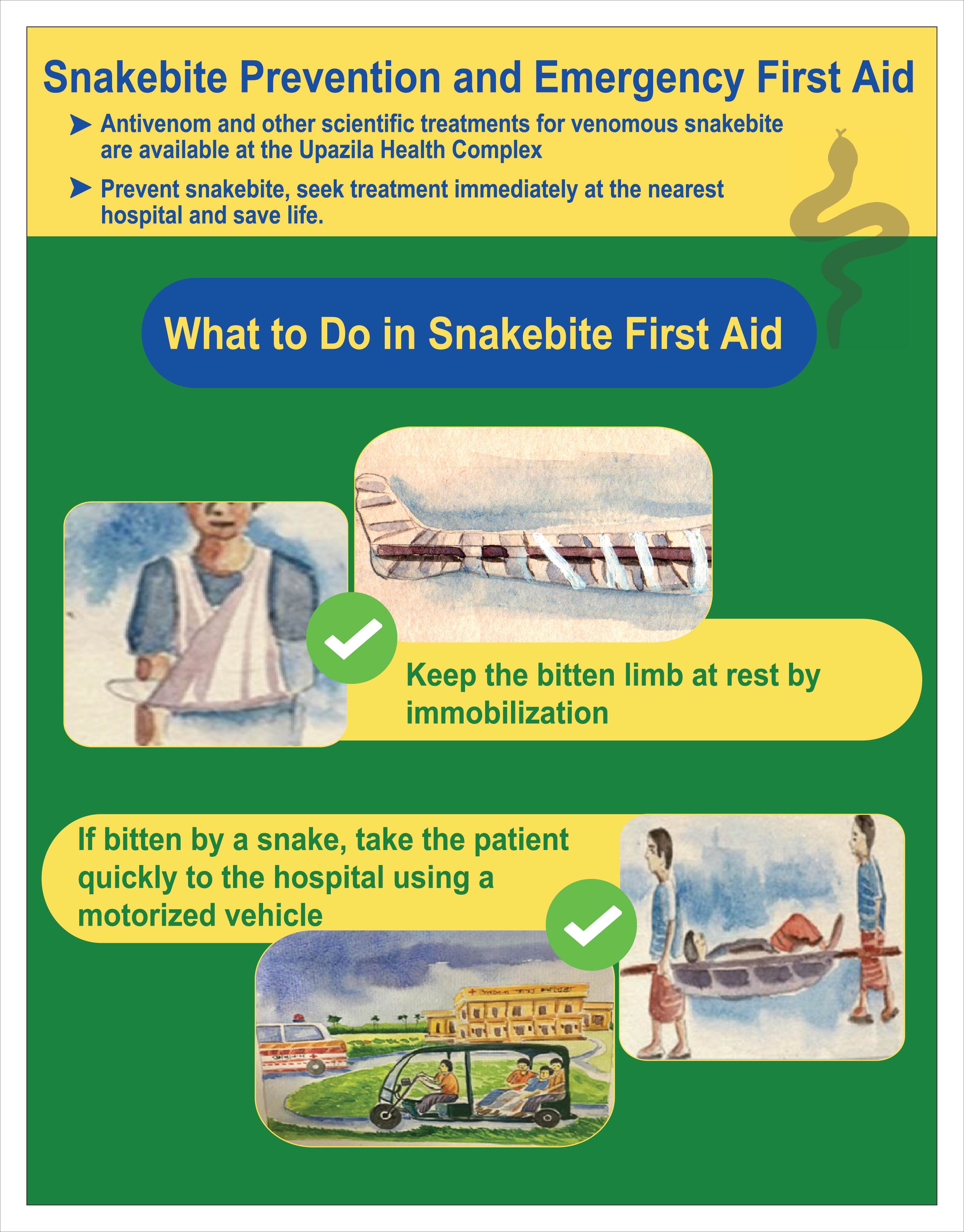

Supplement: S1 Poster — (JPG) [file pntd.0014180.s001.jpg]

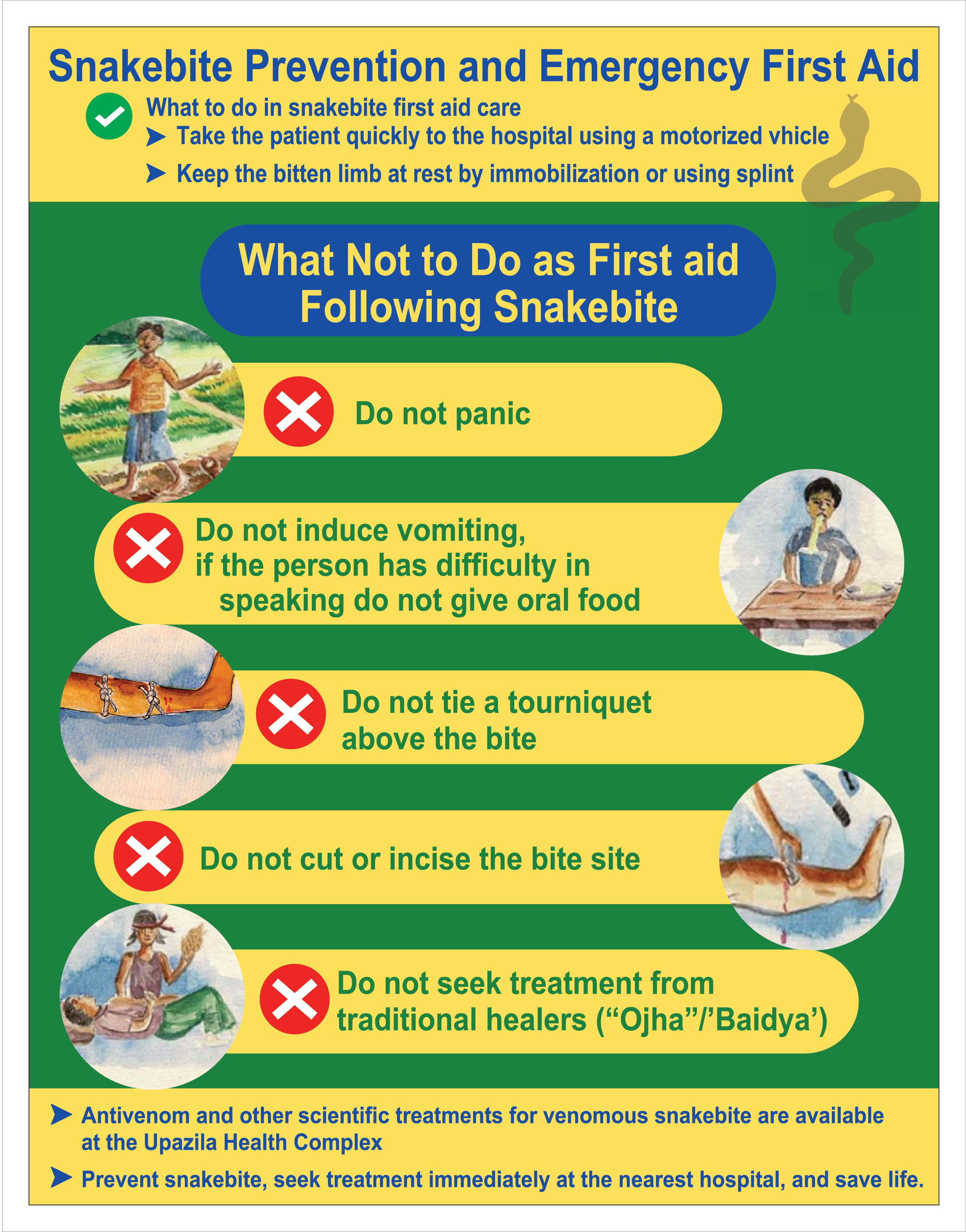

Supplement: S2 Poster — (JPG) [file pntd.0014180.s002.jpg]

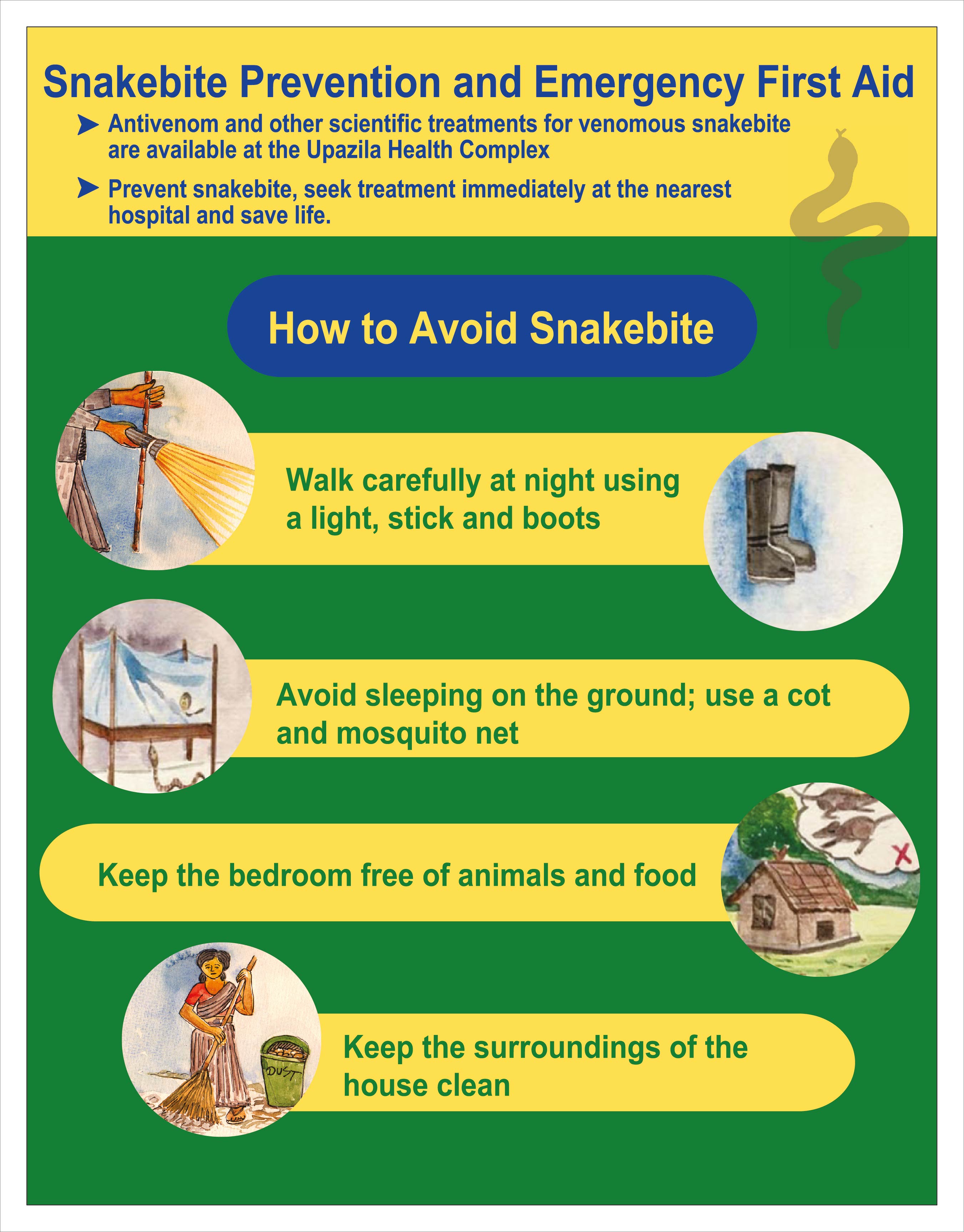

Supplement: S3 Poster — (JPG) [file pntd.0014180.s003.jpg]

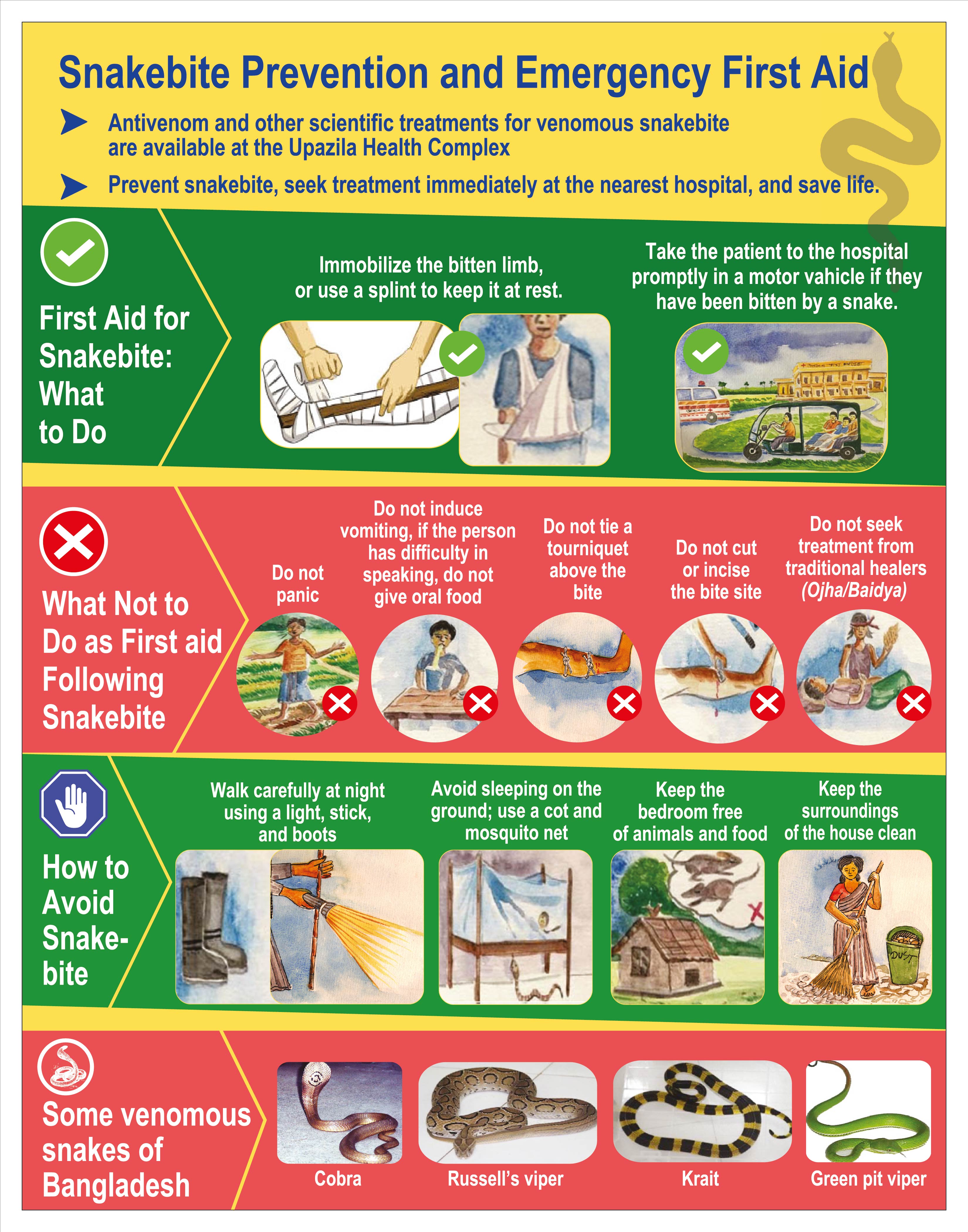

Supplement: S4 Poster — (JPG) [file pntd.0014180.s004.jpg]
