# Supplementary material for: Effectiveness of community engagement in snakebite prevention and proper first aid practices: A community trial in rural Bangladesh
Source: PLoS Negl Trop Dis. 2026 May 18;20(5):e0014180. doi: 10.1371/journal.pntd.0014180 (PMC13262932; doi:10.1371/journal.pntd.0014180)
Supplement: Attachment — Submitted filename: Response to reviewers _30th_sep.docx [file pntd.0014180.s009.docx]

Reviewer #1:

1. **Please, in Figure 2, the population of the Two subdistricts (5,40,0000), intervention (1,98,000) and control area (3,30,0000) should be corrected by either removing the commas or by placing the commas at the desired places. That is either Two subdistricts (540,0000), intervention (198,000) and control area (3,300,000) or Two subdistricts (5400000), intervention (198000) and control area (3300000). In order way, the authors will be in a good position to address this mismatch better.**

*Ans: Thank you for pointing out the inconsistency in in Figure 2. We have corrected the numbers to ensure clarity.*

2. **Please note that the selection of intervention and control areas was not truly random, but “purposive,” which could introduce bias. More detail is needed on how baseline comparability was ensured and how contamination between areas was minimized. Also,** **I noted in the write up that some terms like “design effect of 5.99” are mentioned without sufficient justification or explanation, which may confuse readers who are not versed in complex sampling strategies.**

*Ans: We appreciate the reviewer’s careful observation. As we mentioned in the manuscript, the intervention and control areas were indeed selected purposively, mainly due to operational feasibility and the higher burden of snakebite cases in the intervention site compared to the control site. While Shibganj Upazila was selected as the control site due to its broad socio-demographic comparability and geographic distance, which minimized the likelihood of contamination. To ensure* ***baseline comparability,*** *we used both the* Population and Housing Census 2022*. Census data confirmed that Kalapara and Shibganj were broadly similar in terms of household size, sex ratio, access to electricity, mobile phone ownership, and youth NEET rates.*

*Comparative table of Kalapara and Shibganj on key sociodemographic characteristics*

| ***Indicators*** | ***Kalapara*** | ***Shibganj*** |
| --- | --- | --- |
| *Household size* | *4.00* | *3.9* |
| ***Male: Female*** | ***1.02:1*** | ***1.01: 1*** |
| *Ownership of mobile phone* | *58.89%* | *52.08%* |
| *Access to electricity* | *99.35* | *98.43* |
| *NEET youth population ** | *39.97* | *39.63* |

*Source: population and housing census, 2022*

**Proportion of youth (aged 15-24 years) not in education, employment or training*

*We also took specific steps to minimize contamination between areas, such as choosing geographically distant subdistricts (separated by several hundred kilometers).*

*To avoid the confusion of the readers regarding the design effect, we have deleted that sentence in revised version of manuscript.*

*(Line 150-Line 159 in revised manuscript and Table 1)*

**3. Please, the introduction gives a good overview, but it does not clearly establish the novelty of the current study (what sets this study apart from similar interventions in the region or globally). Kindly consider stating the novelty more explicitly—what specific gaps in the literature does this study fill? Likewise, consider highlighting how the approach employed in this study differs from past approaches in Bangladesh or other countries, which would make the paper’s novelty clearer to readers.**

*Ans: Thank you. We have modified it. (Line 99-110 in revised manuscript)*

4. **Please, if possible conduct a statistical analysis on the most important features contributing to snake bite and discussion the impact of these features accordingly (either positive or negative). This can be done using the logistic regression algorithm which was adopted in the study. Also, figures and tables should be referenced more clearly in the text to guide the reader.**

*Ans: Thank you very much for this insightful suggestion. We agree that logistic regression analysis to identify factors associated with snakebite incidence would provide important evidence on risk and protective determinants. However, we believe that this analysis fits more appropriately within a separate paper we are preparing that specifically focuses on snakebite incidence and case-related predictors. In the present manuscript, our primary aim is to emphasize the effectiveness of community engagement in improving health literacy and preventive practices around snakebite. Therefore, we have kept the scope of statistical analysis focused on knowledge and practice outcomes.*

**5. Please, the limitations section is brief and rather generic. A more thorough reflection on study limitations—such as the risk of reporting bias, the challenges of follow-up, the potential for recall bias in self-reported outcomes, and the limited generalizability due to the selection of only two subdistricts—would make the discussion more robust and trustworthy.**

*Ans:* ***Here is the modified version:*** *This study has important limitations. The* ***intervention and control districts were purposively selected,*** *which may have introduced selection bias and limits the generalizability of findings to other settings with different socioeconomic or ecological characteristics. Especially, the findings may not have been as comprehensive due to the exclusion of children and underrepresentation of diverse occupational groups. Although we accounted for clustering in the analysis, the assumptions regarding the* ***intra-cluster correlation coefficient (ICC), cluster size, and design effect*** *may have influenced the precision of the effect estimates. On the other hand, the study did not follow the same cohort over time but rather employed repeated cross-sectional surveys, which may have influenced the measurement of changes in snakebite-related knowledge. Furthermore, the outcomes were based on self-reported knowledge and practices, making them susceptible to recall and reporting biases that could have resulted in either underestimation or overestimation of the true effects. Despite these limitations, the results provide a scalable framework for improving immediate care and prevention of snakebite in environments with limited resources. (Line 455-466 in revised manuscript)*

**6. Please, the conclusion states that the intervention "should be implemented across Bangladesh and elsewhere," although it provides no discussion on the necessary practical procedures for scaling up, the requisite resources, or the policy consequences. What is required to implement such initiatives on a large scale, and what responsibilities should government, NGOs, or community leaders assume? This section might be enhanced with further actionable advice.**

*Answer: While our findings indicate that community engagement can improve snakebite-related health literacy, scaling up such initiatives must be aligned with Bangladesh’s National Snakebite Strategy. The strategy already outlines a governance structure through the formation of a national steering committee, technical committee, working group, and district- and upazila-level snakebite committees, with clearly defined roles. At the grassroots level, Union Parishads and community clinic groups are mandated to include snakebite prevention and control in their regular meetings. Embedding community engagement interventions within this governance framework would ensure that efforts are not implemented in isolation, but are instead institutionalized and monitored through existing mechanisms. Operationalizing this approach would require the government to strengthen resource allocation and accountability, NGOs to support innovation in awareness and training methods, and community leaders to facilitate local ownership and cultural adaptation.*

*(Line 472-484 in revised manuscript)*

Reviewer #2:

**1. title needs revision, at 1st say effect, then outcome, revise**

*Ans: Thank you for your valuable suggestion regarding the title. We understand that using both “effect” and “outcome” together may sound redundant. To address this, we have revised the title to maintain clarity and consistency. Since our study was a community trial designed to measure the causal impact of the intervention, we have retained the term “Effect” and removed “Outcome.”*

*The revised title:*

*“Effect of community engagement and health literacy on snakebite prevention and proper first aid practices: A community trial in rural Bangladesh.” (Line 2-3 in revised manuscript)*

**2. line 34, …culturally tailored community engagement activities what does this mean?**

*Ans: By ‘culturally tailored,’ we refer to interventions that actively engaged the community and were adapted to the local cultural context, thereby making them more relatable and acceptable to participants. For instance, the script of the community drama was carefully developed to reflect local realities, and popular local actors were chosen to enhance audience identification and trust. In addition, traditional folk singers performed street shows featuring theme-based Boyati songs, a sub-genre of Murshidi or Bhab Gaan rooted in religious and spiritual themes. As Boyati songs are widely appreciated in the Barisal area for their close connection to the community’s cultural and spiritual life, incorporating them ensured that the health messages were delivered through a familiar and respected medium.*

**3. Please provide the supplementary file of statistical analysis for transparency.**

*Ans: Thank you, we have attached the data files as supplementary file.*

**4. Revise abstract to include only key contents**

*Ans: Thank you, we have modified it.*

5. **In the introduction community engagement and health literacy effect not adequately described, even health literacy not mentioned, is it similar with awareness, similar for community engagement, not described, what does it mean, levels of community engagement, link with citizen science as an approach, this issue should be clear**

*Ans: We thank the reviewer for pointing this out. In the revised introduction, we have clarified the concepts of community engagement and explained their relevance to our study in the introduction and methodology section. (Line 99-104 and table 2)*

6. **In sample size determination, what is the base for choosing mentioned effect size? also ICC? How clustering effect is controlled? why attrition rate not considered? how 500 is chosen? which software used to calculate? what is the sampling technique? it is not clear**

*Ans: We thank the reviewer for pointing out the need for clarification. Calculations were performed in and the sampling method followed a multistage cluster design with PPS. The effect size (reduction in prevalence from* ***0.60% to 0.33%****) was chosen based on programmatic expectations of the intervention’s impact after the implementation period, in consultation with experts and stakeholders. A 40-45% relative reduction was deemed both realistic and epidemiologically meaningful for detecting change at the population level. Based on administrative boundaries and logistical feasibility, 500 individuals per cluster represented a reasonable size:*

1. *Large enough to capture within-cluster variability.*
2. *Practical for survey teams to complete within the fieldwork period.*

*The surveys were cross-sectional at baseline and endline (not longitudinal follow-up of the same households). Therefore, attrition of individuals/households was not applicable.*

***Software used****: The initial sample size was calculated using Stata.*

***Sampling technique:*** *A multistage cluster sampling method was used:*

1. *Unions were randomly selected as intervention and control areas in consultation with local authorities.*
2. *Within each union, clusters of households were drawn systematically from census lists.*
3. *Within selected clusters, households were randomly sampled until the target size was reached.*

**7. The design of the study is not clear, why not RCT, justify? is it repeated cross sectional or longitudinal? which is directly linked with statistical analysis you are going to perform? is base and end line population similar or different? clearly describe**

*Ans: Thank you for raising the concern. This study adopted a repeated cross-sectional, quasi-experimental design with intervention and control arms. Independent samples of households were surveyed at baseline and endline using identical strategies, allowing population-level comparisons of snakebite prevalence, knowledge, and practices. We also considered a randomized controlled trial (RCT) design but found it ethically and operationally infeasible in our study context. Barishal, which reports one of the highest snakebite burdens in Bangladesh, was purposively selected as the intervention site to prioritize delivery of the intervention to the most vulnerable population. Randomizing districts or clusters would have meant either (i) excluding Barishal despite its high burden, which we considered ethically unjustifiable, or (ii) randomizing clusters within Barishal, which would have introduced a high risk of contamination due to population mobility and shared health services.*

*Furthermore, implementing a cluster RCT or a stepped-wedge design would have required longer timelines, and additional resources beyond our funding and operational capacity. Under these constraints, purposive selection offered the most pragmatic balance of ethical responsibility, feasibility, and scientific rigor.*

*However, to reduce the selection bias, Rajshahi was selected as the comparison site because it is a large rural district with socio-demographic and health system features broadly comparable to Barishal. It also had no overlapping snakebite programs and is geographically distant (>500 km from Barishal), which eliminated the risk of contamination.*

**8. Include theory of change to show how changes come, assumptions with mediator and moderator variables.**

*Ans: Thank you, we have included it. (Figure 1)*

**9. Figure 2 is not clear, do separately, also it is conceptual framework??**

**Ans:** thank you for pointing out. We modified and renamed the framework as methodological framework.

**10. How fidelity, acceptability, of intervention determined?**

*Ans:* *Fidelity of the intervention was determined through midline process evaluation, including monitoring of planned vs. delivered activities (e.g., number of community meetings, seminars, conducted, and proportion of the target population reached). Supervisors used structured checklists to assess the quality and consistency of intervention delivery.*

*For the assessment of the acceptability, we conducted two focus group discussions, and in-depth interviews with newly snakebite affected victims what caused the barriers/facilitators affected their treatment.*

**11. How tailoring of IEC materials was produced and disseminated? who have done it**

*Answer:* *Information, Education, and Communication (IEC) materials were developed by the research team by a series of workshop with clinicians, public health experts, and communication specialists. To guarantee cultural and contextual relevance, community health workers, educators, and local stakeholders also participated in the creation and presentation of these materials. Content was tailored through formative research, and pre-testing to ensure cultural appropriateness, clarity, and technical accuracy. Focus group discussions and in-depth interviews with community members, traditional healers, and health care workers helped identify misconceptions and locally relevant risk practices. Draft materials (posters, flipcharts, leaflets) were pre-tested among people with similar educational level to check clarity, language, illustrations, and acceptability. Revisions were made based on feedback. Messages were translated into Bangla and visuals were simplified to match literacy levels. The finalized materials were disseminated by field staffs, and facilitators of educational sessions through courtyard meetings, clinics, schools and community gatherings under supervision of project staff.*

**12. How data collection tool is developed its validity, reliability**

*Ans: The data collection tool was developed through literature review and adaptation of existing snakebite KAP questionnaires and WHO guidelines. Items were translated and culturally tailored to the Bangladeshi rural context. Unfortunately, we have not validated the tool.*

**13. I am not convinced with statistical analysis applied, I think difference in difference is appropriate, are population.**

*Ans: We appreciate your thoughtful observation. In our study, both incidence as well as knowledge and practice scores followed non-parallel trends: the intervention group showed sharp declines or improvements, while the control group showed modest increases or stability. So, it violates the parallel trends assumption required for Difference-in-Difference (DID). At baseline, the incidence rate of snakebites was already substantially higher in the intervention area (294.4/100,000) compared to the control area (91.7/100,000). Over time, the intervention area showed a sharp decline (–43.4%), while the control area showed a slight increase (+10.6%). These divergent trajectories indicate that the two areas were not moving in parallel, even before considering the intervention effect.*

**14. There is nothing said about community engagement in method also, which is boldy written on title, similar also for health literacy.**

*Ans: We thank the reviewer for pointing this out. We agree that the methods section did not sufficiently describe the community engagement and health literacy components, despite their prominence in the title. Health literacy was addressed by delivering structured educational sessions on snakebite prevention and first aid, complemented by behavior change communication (BCC) materials.*
