# Supplementary material for: Effectiveness of community engagement in snakebite prevention and proper first aid practices: A community trial in rural Bangladesh
Source: PLoS Negl Trop Dis. 2026 May 18;20(5):e0014180. doi: 10.1371/journal.pntd.0014180 (PMC13262932; doi:10.1371/journal.pntd.0014180)
Supplement: Attachment — Submitted filename: Response to Reviewers_2nd revision_January 13.docx [file pntd.0014180.s010.docx]

**Response to Reviewers**

**Reviewer 1:**

The authors have addressed some of the significant comments raised in my previous review.

*Response: Thank you very much.*

**Reviewer 2:**

Point 1: Revise title, is it effect or effectiveness ? In discussion, you used impact of

Please use appropriate which can contain community engagement and health literacy ? through community engagement you can raise health literacy, it might be. Effectiveness of community engagement through on snakebite prevention and proper………. First aid practices: A community trial in rural Bangladesh.

*Response: ‘The Title’ has been revised according to your suggestion. Now the title of the manuscript is “Effectiveness of community engagement in Snakebite Prevention and Proper First aid Practices: A community trial in rural Bangladesh.” (Line 2 and 3)*

Point 2:

What does culturally tailored community engagement activities mean ? Use appropriate term from the levels of community engagement from informing to empowering which is implemented in your study? Clearly explain the methods the levels, how it was done, challenges encountered, limitation acknowledged.

*Response:*

Revised as per instruction.

By ‘culturally tailored,’ we refer to interventions that actively engaged the community and were adapted to the local cultural context, thereby making them more relatable and acceptable to participants. For instance, the script of the community drama was carefully developed to reflect local realities, and popular local actors were chosen to enhance audience identification and trust. In addition, traditional folk singers performed street shows featuring theme-based Boyati songs, a sub-genre of Murshidi or Bhab Gaan rooted in religious and spiritual themes. As Boyati songs are widely appreciated in the Barisal area for their close connection to the community’s cultural and spiritual life, incorporating them ensured that the health messages were delivered through a familiar and respected medium.

**The details of community engagement that we followed has been included in the method section. In the community engagement we included various levels like inform, consult, involve, and empower the community. However, we have revised the texts as per instruction. (Line 202 to line 207 and table 2 )**

**The main challenges we encountered during community engagement process are 1. Resources and time constraints, 2. Managing expectation and conflicting priorities, and 3. Superficial engagement.**

**Limitation has been discussed in the discussion sections. (Line 469 to 471 )**

Point 3:

In sample size what is the evidence to use 40%, is clustering not needed?

*Response:*

a) *The effect of community engagement and health outcome varied. There is evidence that the high community engagement was associated with the rate of differences approximately 49% in vaccination uptake.( Xie J., Liao X., Lin M., et al. (2024). Community engagement in Vaccination promotion: systematic review and meta analysis. JMIR Public Health and Surveillance, 10, e49695.) Another meta-analysis (BMC Public Health, 2015) on community engagement interventions across health outcome reported that the overall effect size for health behavior outcomes 33%. This also provides a robust, peer-reviewed benchmark around 30%-40% range for effects of engagement-oriented programs. ( O’Mara-Eves A., Brunton G., Olover S., et al. (2015). The effectiveness of community engagement in public health interventions for disadvantaged groups: a meta-analysis. BMC Public Health, 15, 129.) The reference has been inserted. (Line 171, Reference 30-31).*
b) *For the intervention, we did not need clustering since all population of selected communities was included in the study, and population was homogeneous.*

Point 4:

How you reach consensus on IEC development and then proceed to implementation, what representative population used in pretesting, where is the site of pretest, how you select the IEC materials.

*Response:*

*We developed a communication strategy in collaboration with communication specialists and conducted a series of workshops with diverse stakeholders to guide the selection, design, and production of IEC materials for the research project. Once the materials were developed, they were pre-tested in communities similar to the intervention area. Feedback from this pre-testing was then incorporated to finalize the IEC materials. The detail process of IEC material development has been presented in methods section. (Line 219 -line 231)*

Point 5:

How you measured health literacy?

*Response: Health literacy was measured utilizing population-level toos which were culturally adapted, language validated, and education level sensitive. The following outcome variables were considered during measurement of health literacy: Knowledge on preventive measure, knowledge on First aid, and Practice. (Line 258-line 267 and table 3)*

Point 6:

The theory of change is not clear, what were the assumptions, the indicators, the chain of outcomes?

*Response: Revised according to the instruction. We have included the assumptions, and indicators.*

Point 7:

I don’t see any community engagement concept in your study, …

*Response: Community engagement concept has been added in the introduction section as well as in Methods section. We used The Arnstein’s Ladder of citizen participation” for ensuring community engagement. (line 99-line 111 and Line 232 To 235)*
